# RelB promotes liver fibrosis via inducing the release of injury‐associated inflammatory cytokines

**DOI:** 10.1111/jcmm.15108

**Published:** 2020-04-19

**Authors:** Danhua Zhou, Wei Huang, Jinhuan Wei, Jingxin Zhang, Zhaoxiu Liu, Ran Ji, Sijia Ge, Mingbing Xiao, Yihui Fan, Cuihua Lu

**Affiliations:** ^1^ Department of Gastroenterology Affiliated Hospital of Nantong University Jiangsu China; ^2^ Laboratory of Medical Science School of Medicine Nantong University Jiangsu China; ^3^ Department of Pathogenic Biology School of Medicine Nantong University Jiangsu China; ^4^ Department of Gastroenterology and Research Center of Clinical Medicine Affiliated Hospital of Nantong University Nantong China

**Keywords:** hepatocyte, inflammatory cytokines, Liver fibrosis, NF‐kB, RelB

## Abstract

Liver fibrosis is a serious chronic disease that developed by a coordinated interplay of many cell types, but the underlying signal transduction in individual cell type remains to be characterized. Nuclear factor‐κB (NF‐κB) is a widely accepted central player in the development of hepatic fibrosis. However, the precise role of each member of NF‐κB in different cell type is unclear. Here, we generated a mouse model (*Relb* ^Δhep^) with hepatocyte‐specific deletion of RelB, a member of NF‐κB family. *Relb* ^Δhep^ mice born normally and appear normal without obvious abnormality. However, in the CCl4‐induced liver fibrosis, *Relb* ^Δhep^ mice developed less severe disease compared with wide‐type (WT) mice. The denaturation and necrosis of hepatocytes as well as the formation of false lobules in *Relb* ^Δhep^ mice were significantly reduced compared with WT mice. The production of α‐SMA and the level of collagen I and Collagen III were greatly reduced in *Relb* ^Δhep^ mice comparing with WT mice. Furthermore, in patients with liver fibrosis, RelB is up‐regulated along with the stage of diseases. Consistently, CCl4 treatment could up‐regulate the expression of RelB as well as inflammatory cytokines such as IL‐6 and TGF‐β1 in hepatoma cell as well as in WT mice. Knockdown the expression of RelB in hepatoma cells greatly reduced the expression of CCl4‐induced inflammatory cytokines. In summary, we provide the genetic evidence to demonstrate the critical and hepatocellular role of RelB in liver fibrosis. RelB is an important transcription factor to drive the expression of inflammatory cytokines in the initiation phase of injury.

## 1 INTRODUCTION

Hepatic fibrosis is a reversible wound‐healing response that occurs in almost all patients with chronic liver injury caused by a variety of reasons such as chronic viral infection, alcoholic liver disease, non‐alcoholic steatohepatitis and autoimmune hepatitis.^1^, ^2^ Hepatic fibrosis is characterized by the continuous deposition of extracellular matrix (ECM), which is mainly derived from the activated hepatic stellate cells (HSCs).^3^ As the most abundant cell in the liver, the damaged hepatocyte could release numerous cytokines and cell contents, thereby promotes the transformation of HSCs into myofibroblasts.^4^ As the disease progress, the terminal stage of hepatic fibrosis can develop into cirrhosis or even hepatocellular carcinoma (HCC),^5^ it is an urgent need to figure out the mechanisms of fibrogenesis.

As a key transcriptional regulator of the inflammatory response, nuclear factor‐κB (NF‐κB) has a wide range of functions in the progression of liver fibrosis,^6^ such as functions involved in the survival of hepatocytes and the activation of HSCs. NF‐κB family includes NF‐κB1 p50/p105, NF‐κB2 p52/p100, RelA/p65, RelB and c‐Rel.^7^ Based on the components of the signalling cascade, NF‐κB signalling pathway could be divided into canonical and non‐canonical pathway.^8^ The canonical nuclear factor‐kappa B (NF‐κB) pathway is triggered by immune signals that activate the kinase TGFβ‐activated kinase 1 (TAK1). Then, TAK1 activates the trimeric IκB kinase (IKK) complex via phosphorylation of IKKβ. Upon stimulation, the IKK complex phosphorylates IκBα and p105, resulting in the ubiquitin (Ub)‐dependent degradation of IκBα and p105, ultimately leading to nuclear translocation of classical NF‐κB family members, including RelA‐p50, c‐Rel‐p50 and p50‐p50.^7^ By contrast, non‐canonical NF‐κB signalling mainly regulates RelB by the processing of p100. The non‐canonical NF‐κB pathway selectively responds to the tumour necrosis factor receptor (TNFR) superfamily members, which activates NF‑κB‑inducing kinase (NIK).^9^ NIK phosphorylates and activates IKB kinase alpha (IKKα), which leads to the phosphorylation and selective degradation of p100, thereby enabling nuclear translocation of p52/RelB heterodimers.^10^ However, as a member of the NF‐κB family, the molecular mechanisms by which RelB promotes hepatocyte injury and hepatic fibrosis remain poorly understood. During the preparation of this manuscript, a recent reported study shows critical function of RelB in cholangiocyte response to injury as well as biliary fibrosis.^10^ However, the role of Relb in hepatocyte‐driven fibrosis remains to be addressed.

In this study, we generated a mouse model (*Relb* ^Δhep^) with hepatocyte‐specific deletion of RelB. We aimed to explore the roles of RelB in the injury of hepatocyte, the activation of HSCs and the progression of fibrosis in vivo and in vitro. Our study may provide new insights into the mechanisms underlying hepatic fibrogenesis.

## 2 MATERIALS AND METHODS

### 2.1 Animals

All mice were propagated in the background of the C57BL/6 gene. Albumin‐Cre mice were purchased from Shanghai Model Organisms. Relb^loxp/loxp^ mice with a loxP‐flanked Relb allele on a C57BL/6J background were purchased from The Jackson Laboratory. (Jax:0287, B6.Cg‐Relbtm1Ukl/J). Albumin‐Cre mice were crossed with Relb^loxp/loxp^ mice to produce Relb^fl/fl^/Alb‐Cre mice. Mice were housed in the environment of 12 hour light/dark with constant temperature. Six‐ to eight‐week‐old female mice were used to establish hepatic fibrosis model by injection of carbon tetrachloride (CCl4, 5 mL/kg bodyweight, 1:4 dilution in olive oil), twice a week for 5 weeks. All the animal experiments were approved by the animal experimentation ethics committee of the Affiliated Hospital of Nantong University.

### 2.2 Haematoxylin and eosin staining

Liver tissues were fixed in 4% paraformaldehyde fix solution for 24 hours and embedding with paraffin, in order to investigate mouse liver histology. Five micrometer thick sections were deparaffinized and hydrated to water, and then stained with haematoxylin for 20 minutes and eosin for 10 seconds (H&E, #E607318‐0200; BBI Life Sciences). Rapid dehydration, transparency and sealing.

### 2.3 Sirius red and Masson staining

Sirius red (G1018, servicebio) and Masson‐Fontana staining (G1057, servicebio) were used to detect collagen deposition. Positive areas were quantified using ImageJ software. Random sections from each mouse were analysed.

### 2.4 Immunohistochemical staining

Immunohistochemistry was performed on 5‐μm thick paraffin sections, with antibodies against α‐SMA (#sc‐32251; Santa cruz), collagen I (#ab34710; Abcam) and collagen III (#ab7778; Abcam). Briefly, 5‐μm thick sections were deparaffinized in xylene and hydrated to water. The endogenous peroxidase activity was blocked by immersing the sections in 3% hydrogen peroxide for 20 min after antigen retrieval. The sections were blocked with 10% goat serum for 1 hour at room temperature and covered with primary antibody for 4°C overnight, followed by incubation with enzyme‐labelled anti‐mouse/rabbit IgG polymer for 30 minutes at room temperature. DAB staining was used to visualize positive staining, and haematoxylin was used for the staining of nucleus. Stained areas were quantified using ImageJ software.

### 2.5 Quantitative real‐time PCR

Total RNA was purified from liver tissues, according to the manufacturer's instructions, and reverse transcribed by Revert Aid First Strand cDNA Synthesis Kit (Thermo). SYBR Green PCR Kit (Qiangen) was used to quantitative the expression of gene. mRNA expression was normalized against GAPDH. The primers of human RelB, IL‐6, TGF‐β1 and GAPDH were designed by primer design program (Primer 3 software version 1.0). The sequences of the primers are shown as follows: Human RelB: 5′‐ATGGCATCGAGAGCAAAC‐3′ (forward); 5′‐AGAGAAGAAGTCAGG‐GTCTG‐3′ (reverse); Human IL‐6:5′‐TGCGTCCGTAGTTTCCTTCT‐3′ (forward); 5′‐GCCTCAGACATCTCCAGTCC‐3′ (reverse); Human TGF‐β1:5′‐GCGTGCTAATGGTGGAAAC‐3′ (forward), 5′‐CGGTGACATCAAAA‐GATAACCAC‐3′ (reverse); Human GAPDH:5′‐TGCACCACCAACTGCTTAGC‐3′ (forward), 5′‐GGCATGGACTGTGGTCATGAG‐3′ (reverse).

### 2.6 Cell culture and CCl4‐induced cell injury model

SMMC‐7721 cells were cultured at humidified 37°C with a 5% CO_2_ atmosphere in high‐glucose DMEM supplemented with 10% FBS (Gibco), 100 U/mL penicillin and 100 μg/mL streptomycin. Before treated, SMMC‐7721 cells were seeded in six‐well plate overnight. Four millimolar CCl4 and DMSO were added to cells for 6 hours to induce cell injury. Protein and mRNA were extracted and detected by RT‐PCR and Western blot.

### 2.7 Cell transfection

Small interference RNAs (siRNA) were purchased from Genechem (Shanghai). The siRNA sequences used in this study are as follows: RelB‐Homo‐274 siRNA: 5′‐CCAGGAGCACAGAUGAAUUTT‐3′, RelB‐Homo‐853 siRNA: 5′‐GGAAGAUUCAACUGGGCAUTT‐3′, RelB‐Homo‐1252 siRNA: 5′‐CCGUGACAGUCAACGUCUUTT‐3′, Negative control siRNA: 5′‐UUCUCCGAACGUGUCACGUTT‐3′. SMMC‐7721 cells were seeded in six‐well plate the day before transfection. Cells were transfected using Lipofectamine 2000 transfection reagent (Invitrogen) according to the manufacturer's instructions. Transfected cells were used for the subsequent experiments 48 hours after transfection.

### 2.8 Western blot analysis

Briefly, cell samples were homogenized in a lysis buffer and centrifuged at 10 000 *g*, 4°C for 15 minutes to collect the supernatant. Protein concentrations were determined by a BCA protein assay kit (Bio‐Rad). Subsequently, the supernatant was diluted in 5× SDS loading buffer and boiled for 15 minutes. The protein samples were separated with 12% SDS‐polyacrylamide gel electrophoresis (SDS‐PAGE) and transferred to polyvinylidene difluoride filter (PVDF) membranes (Millipore), then, blocked the membranes with 5% non‐fat milk in TBST for 2 hours and incubated with primary antibodies overnight at 4°C. Thereafter, the membranes were incubated with the secondary antibody horseradish peroxidase‐linked IgG for 2 hours. The detection of chemiluminescent signals was performed by ECL method (ZhongShan Biotech Company).

### 2.9 Statistical analysis

Results are expressed as means ± SEM. Statistical analysis of values was performed by two‐sided independent Student's *t* test. Statistical significance was indicated as: *P < .05; **P < .01.

## 3 RESULTS

### 3.1 Hepatocyte‐specific RelB knockout inhibits hepatocyte injury and hepatic fibrogenesis in vivo

**Figure 1:**
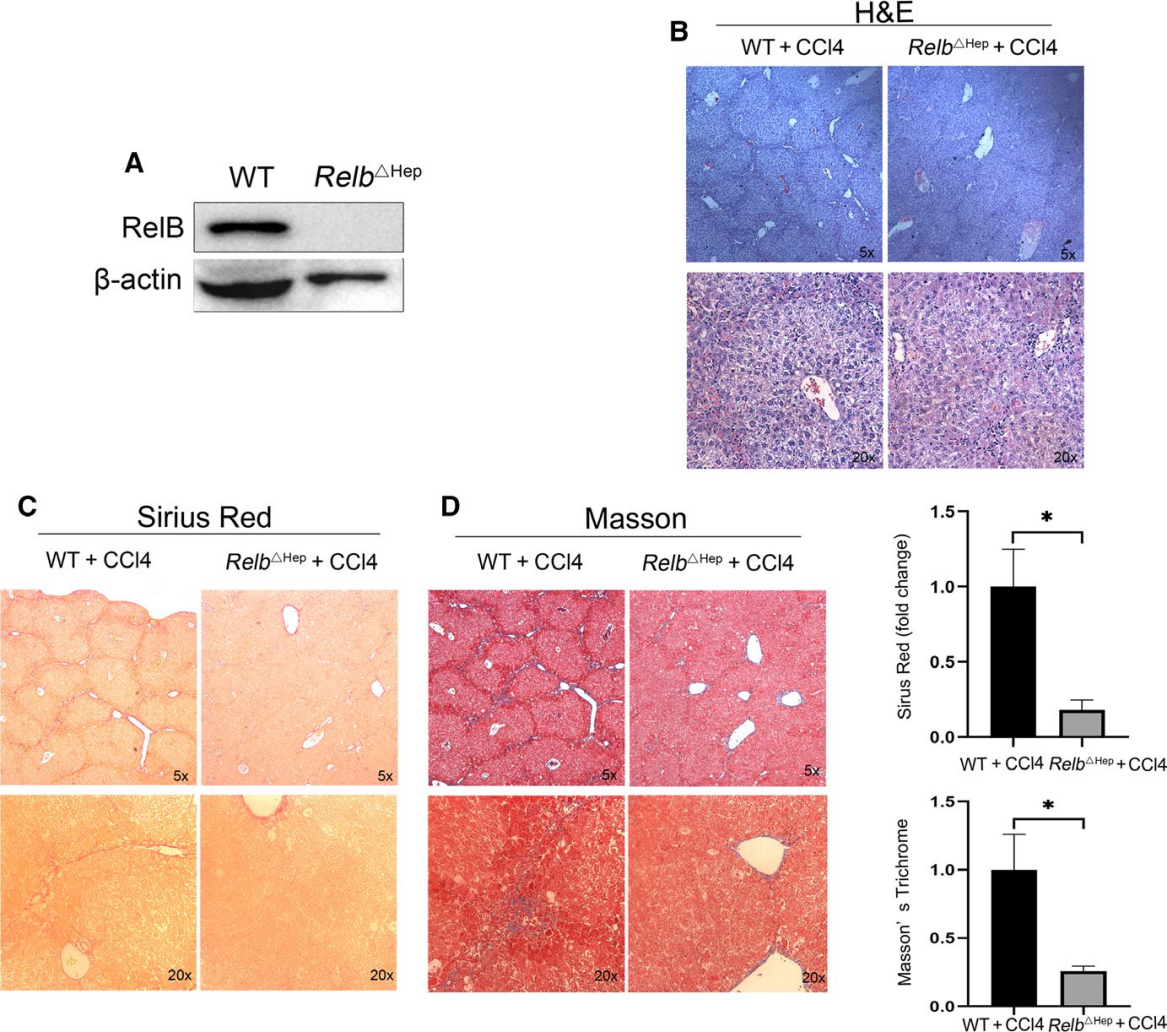
Hepatocyte‐specific RelB knockout inhibits progression of liver fibrosis. (A) Western blot assay analysed the expression of RelB in primary hepatocytes isolated from *Relb* ^Δhep^ mice and WT mice. Liver sections of 5 μm were stained with haematoxylin and eosin (H&E) (B), Sirius Red staining (C) and Masson's trichrome (D), and scanned with LeicaDM4000B upright microscope.*P < 0.05, **P < 0.01 and ***P < 0.01 indicate a significant difference between the groups

To determine the role of RelB in hepatic fibrogenesis, we successfully created the hepatocyte‐specific RelB knockout mice (*Relb* ^Δhep^). Primary hepatocytes isolated from WT and *Relb* ^Δhep^ mice were used to confirm the deletion of RelB by WB (Figure 1A). *Relb* ^Δhep^ mice born normally and appear normal without obvious abnormality (data not shown). Next, we induced the liver fibrosis both in WT and *Relb* ^Δhep^ mice by CCl4. As shown in Figure 1B, 5 weeks after CCl4 injection, the architecture of liver in WT mice was greatly distorted. The hepatocytes show severe denaturation and marked necrosis as well as the formation of false lobules in WT mice (Figure 1B). However, we found that the architecture of liver in *Relb* ^Δhep^ mice was gently distorted, and the hepatocyte was only slightly damaged and the false lobules were rarely formed (Figure 1B). Furthermore, the Sirius Red staining and Masson's Trichrome show a significant decrease of collagen deposition in *Relb* ^∆hep^ mice than that in WT mice (Figure 1C, D). These findings implied that deletion of RelB could attenuate hepatic fibrogenesis in vivo.

### 3.2 *Relb* ^∆hep^ mice have fewer activated hepatic stellate cell and lower ECM deposition than the WT mice

**Figure 2:**
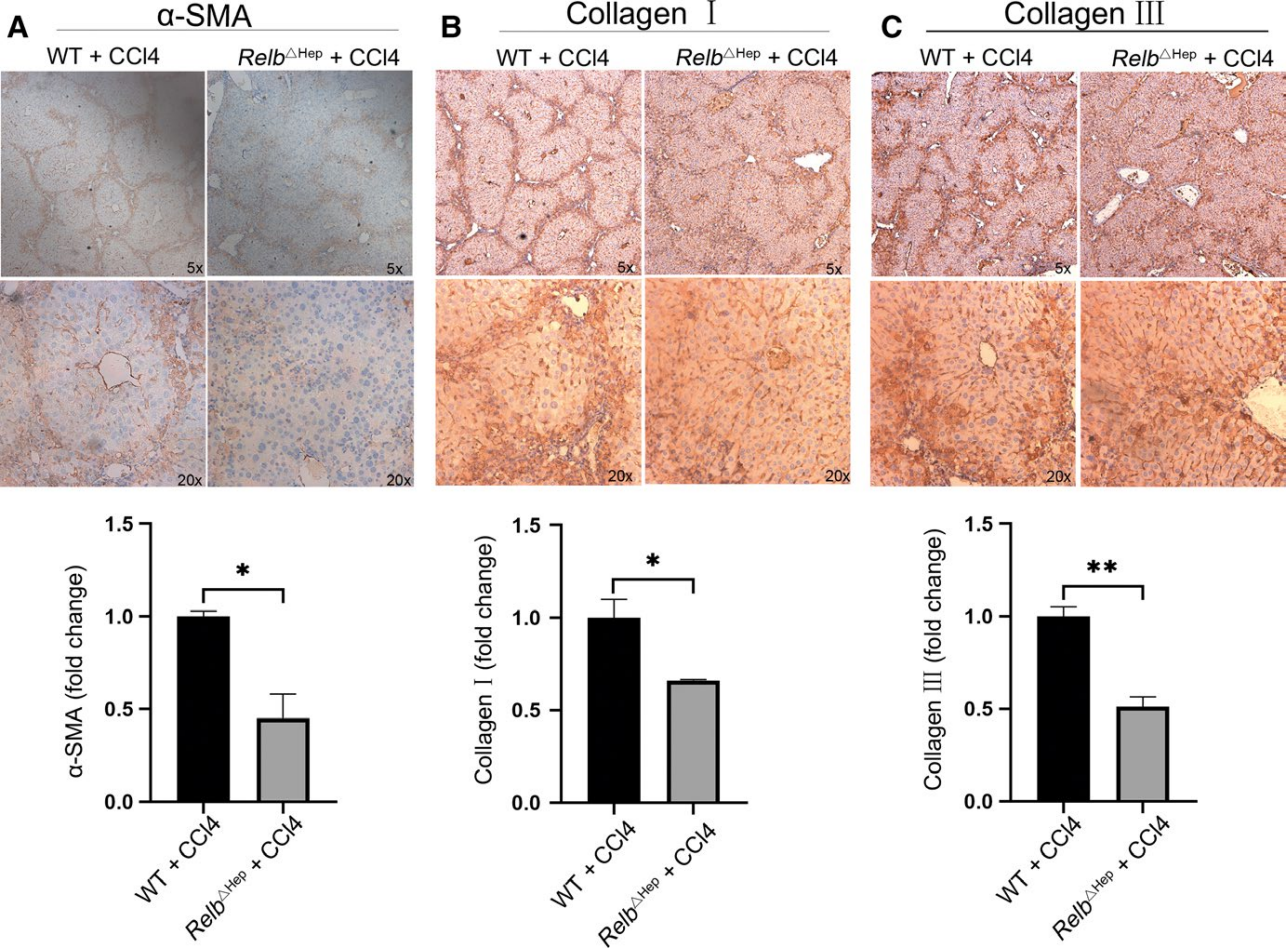
Hepatocyte‐specific RelB knockout mice have lower hepatic stellate cell activation than WT mice. (A) Immunohistochemistry staining on paraffin sections for α‐SMA of WT and *Relb* ^Δhep^ livers after 5 wk CCl4 injection. Immunohistochemistry was performed on paraffin sections for collagen type I (B) and collagen type III (C) in livers of WT and *Relb* ^Δhep^ livers after CCl4 injection.*P < 0.05, **P < 0.01 and ***P < 0.01 indicate a significant difference between the groups

As the most abundant cells in the liver, hepatocytes play prominent roles in the activation of HSCs, which is a pivotal event in the liver fibrotic pathogenesis.^11^ Thus, we detected the expression of α‐SMA, a marker of HSCs activation, and found that it was markedly reduced in *Relb* ^Δhep^ mice compared with WT mice, indicating that the activation of HSCs was suppressed in *Relb* ^Δhep^ mice (Figure 2A). Once activated, HSCs turn into myofibroblasts, resulting in the accumulation of ECM by up‐regulating gene expression.^12^, ^13^ In *Relb* ^Δhep^ fibrosis model mice, the expression of collagen I, which is postulated to be the major component of fibrosis, was lower than that in WT mice induced by CCl4 injection (Figure 2B). Similar trend has been found in the expression of collagen III (Figure 2C). These findings suggested that loss of RelB in hepatocyte is able to ameliorate HSCs activation in CCl4‐induced fibrosis model.

### 3.3 RelB is up‐regulated with fibrosis stages in patients, and it can be significantly up‐regulated upon injury in hepatocytes

**Figure 3:**
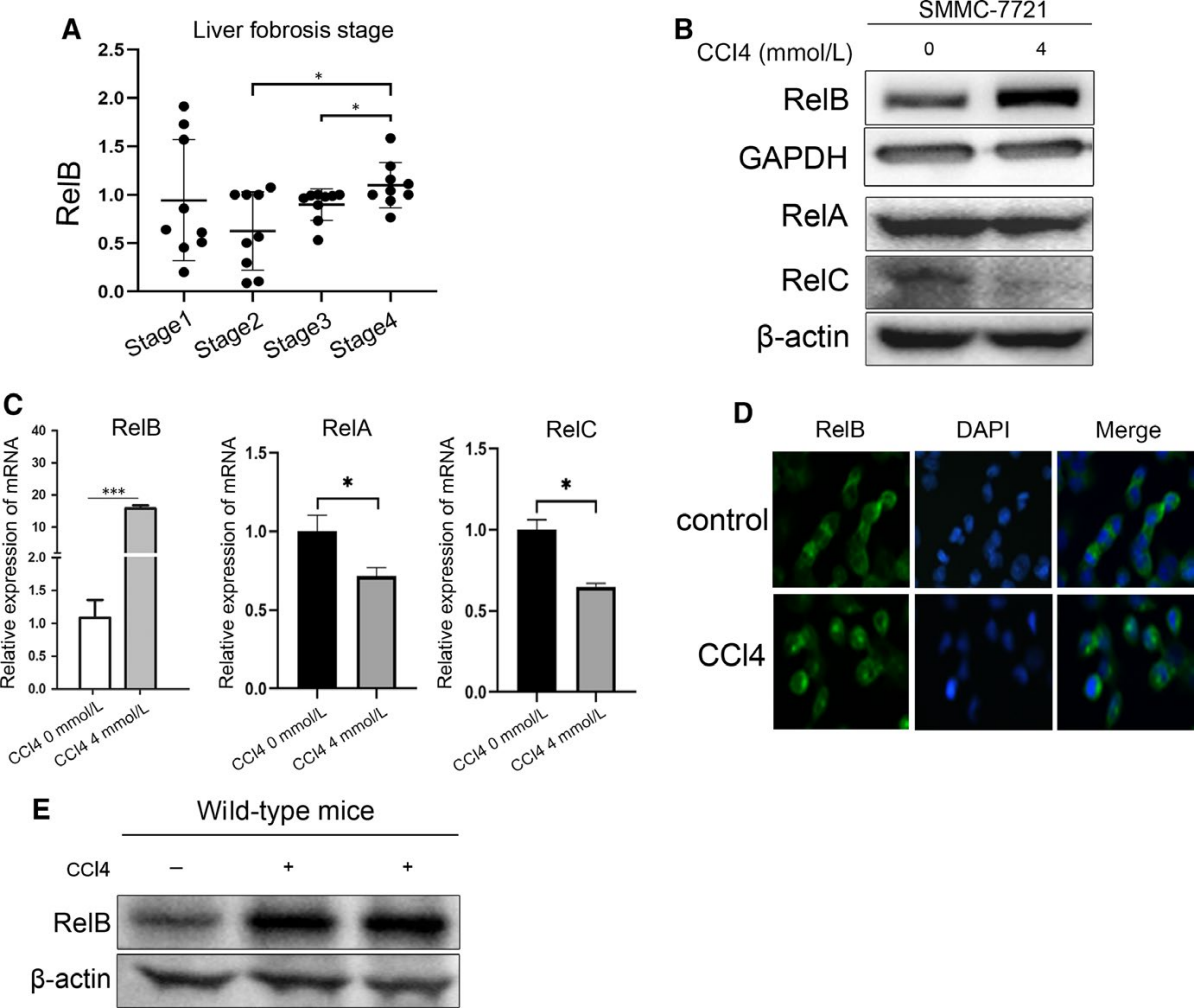
RelB was up‐regulated upon CCl4‐induced injury. (A) A scatter dot plot showing RelB expression levels in patients with four stages of liver fibrosis based on published RNA‐seq data sets. Information for data sets was downloaded from the GEO database (GSE33258). (B) Western blot assay analysed the expression of RelB, RelA and RelC in SMMC‐7721 cells treated by 4 mM CCl4 for 6 h. (C) mRNA expression analysis of RelB, RelA and RelC was quantified by RT‐PCR of SMMC‐7721 treated with or without 4 mM CCl4 for 6 h. (D) Immunofluorescence was used to detect the RelB distribution in SMMC‐7721 cells treated with or without 4 mM CCl4. (E) Western blot assay analysed the expression of RelB in WT mice treated with or without CCl4.*P < 0.05, **P < 0.01 and ***P < 0.01 indicate a significant difference between the groups

Due to the critical role of RelB in mouse model, we examined the relevance of RelB in patients with liver fibrosis. We firstly analysed the expression of RelB in different fibrosis stages from patients with liver fibrosis. Based on the GEO dataset (GSE33258), we found that the expression of RelB was increased with the progress of fibrosis (Figure 3A). It suggests that RelB may play an important role in the progression of liver fibrosis in patients. We further confirmed the change of RelB upon injury, and we treated hepatoma cells with CCl4 to induce injury in vitro. Consistently, CCl4 treatment could greatly up‐regulate the expression of RelB both in mRNA and protein level (Figure 3B, C). However, the other two NF‐κB members (RelA and RelC) were slightly or significantly decreased upon CCl4 treatment, respectively (Figure 3B, C). CCl4 treatment could induce the nuclear translation of RelB (Figure 3D). Similarly, the expression of RelB in WT mice treated with CCl4 was increased than that in WT mice without CCl4‐treated (Figure 3E). Our results demonstrate a potential relevance of RelB in patients with liver fibrosis, and RelB is up‐regulated upon injury.

### 3.4 RelB is required for injury‐induced production of inflammatory cytokines

**Figure 4:**
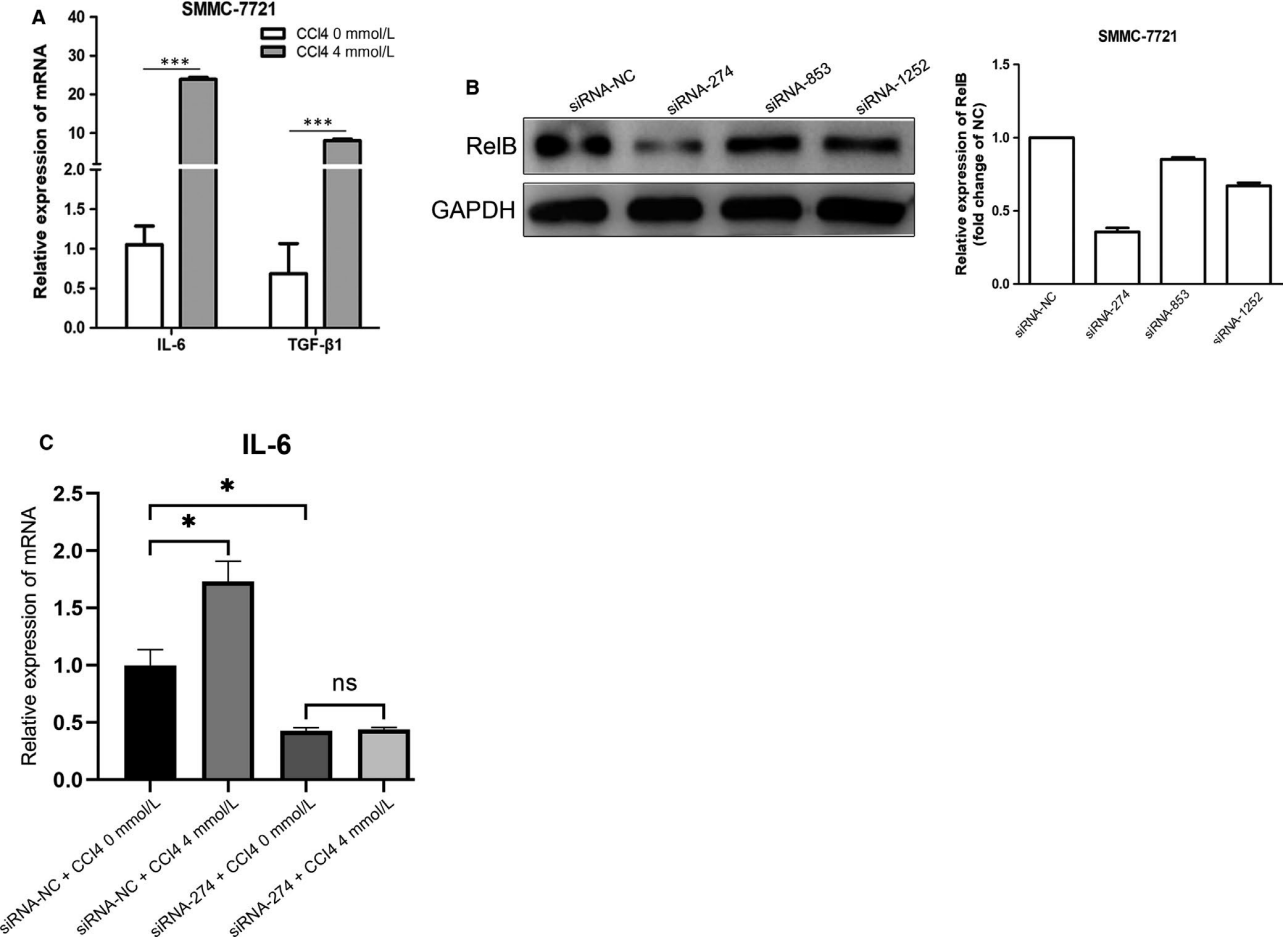
Knockdown RelB reduced the levels of inflammatory cytokines induced by CCl4. (A) mRNA levels of IL‐6 and TGF‐β1 in SMMC‐7721 cells treated with or without 4 mM CCl4 for 6 h. (B) SMMC‐7721 cells were transfected with siRNAs targeting on RelB and control siRNAs. Western blot analyses confirm siRNA‐mediated knockdown of RelB in SMMC‐7721 cells. (C) mRNA expression of IL‐6 in RelB knockdown cells and control cells was examined by RT‐PCR.*P < 0.05, **P < 0.01 and ***P < 0.01 indicate a significant difference between the groups

It is well known that NF‐κB plays critical role in the regulation of inflammatory cytokines. The up‐regulation of RelB in injury indicates that RelB might play critical role in injury‐induced inflammatory. To test this possibility, we treated hepatoma cells with CCl4 and examined the production of inflammatory cytokines. As shown in Figure 4A, CCl4 treatment could significantly induce the expression of inflammatory cytokines such as IL‐6 and TGF‐β1 (Figure 4A). To determine the role of RelB in injury‐induced production of inflammatory cytokines, we knockdown the expression of RelB in hepatoma cells. We found siRNA‐274 could efficiently reduce the expression of RelB (Figure 4B). Knocking down the expression of RelB almost completely abolishes the CCl4‐induced expression of IL‐6 (Figure 4C). Collectively, we demonstrated a critical role of RelB in injury‐induced expression of inflammatory cytokines.

## 4 DISCUSSION

The development of liver fibrosis is thought to be an excessive wound‐healing response, a malignant cycle caused by hepatocyte necrosis, inflammatory response and excessive ECM deposition. Long‐term exposure to virus, alcohol and other risk factors can lead to hepatocyte injury and apoptosis,^14^ then triggers a chronic injury repair mechanism that causes inflammation and ECM deposition, leading to liver fibrosis. The ECM is mainly produced by activated HSCs.^15^ Damage and apoptosis in hepatocytes stimulate HSCs activation and collagen secretion by releasing damage‐associated reactive oxygen species (ROS) and recruiting immune cells.^16^ However, the mechanism of liver fibrosis remains largely unknown.

Although NF‐κB has a wide range of functions in the progression of liver fibrosis, the relationship between non‐canonical NF‐κB signalling and hepatic fibrosis remains largely elusive. As a transcription factor of non‐canonical NF‐κB signalling, Toni Urbanik et al^17^ have found that RelB is significantly activated in CYLD^FF^ *xAlbCre* mice compared with WT mice, and found that RelB is required for the ductular reaction and the progression of biliary fibrosis in this model mice.^10^ They also found that there are no significant differences in fibrosis induced by CCl4 between control, *Relb* ^△LPC^, *Cyld* ^△LPC^ and *Cyld/Relb* ^△LPC^ mouse models. Surprisingly, through constructing RelB hepatocyte‐specific knockout (*Relb* ^△hep^) mouse model, we found that knockout of RelB could protect experimental hepatic fibrosis in mice. In our liver fibrosis model induced by CCl4 injection, H&E staining results shown that hepatocyte was markedly damaged and necrosis in WT mice compared with *Relb* ^△hep^ mice. The Sirius red staining and Masson staining showed that knockout of RelB could significantly attenuate the degree of liver injury and inhibitor the development of hepatic fibrosis in *Relb* ^△hep^ livers compared with WT livers. These results suggested that loss of RelB in hepatocyte protected the injury of liver and inhibited liver fibrosis. In addition, it is widely acknowledged that the composition of ECM in the fibrotic liver was both quantitative and qualitative differences compared with the normal liver.^18^ Thus, we further detected the expression of α‐SMA, the hallmark of HSCs activation and the expression of collagen I and III, the main component of ECM, and found that the expression of α‐SMA, Collagen I and III was markedly reduced in *Relb* ^△hep^ mice than that in WT mice. Taken these results together, we found that knockout of RelB in hepatocyte could reduce the degree of liver injury, inhibit the activation of HSCs, reduce the deposition of ECM, thereby attenuate hepatic fibrosis.

Inflammation is an important component of the healing response to liver injury, and the chronic inflammation is closely related to the development of fibrosis. Previous studies have shown that NF‐κB plays a crucial role in the inflammatory of liver fibrosis.^6^, ^19^ As a target gene of NF‐κB, IL‐6 exerts a crucial role in the regulation of inflammation.^20^, ^21^ IL‐6 could promote the apoptosis of activated HSCs by activating NF‐κB‐inducible nitric oxide synthase signalling.^22^ The HLF/IL‐6/STAT3 feed forward circuit could induce the activation of HSCs, resulting in liver fibrosis.^23^ Thus, in order to explore the underlying mechanisms of loss of RelB reduces liver injury and ameliorates HSCs activation, CCl4 was used to construct cell injury model in SMMC‐7721 cells. Interestingly, we found that the expression of RelB was up‐regulated in SMMC‐7721 cells stimulated by CCl4, accompanied by the increase of IL‐6 and TGF‐β1. To further confirm the regulation of RelB in the levels of IL‐6, siRNA was used to knockdown the expression of RelB in SMMC‐7721 cells. We found that knockdown RelB significantly reduced the levels of IL‐6. This result explained that RelB activates HSCs by promoting the release of inflammatory factors and pro‐fibrotic factors in injured hepatocyte, thereby promoting inflammatory response and ECM deposition, ultimately leading to the progression of liver fibrosis. However, the mechanism of RelB promotes the production of inflammatory factor is unclear, and we will further explore the mechanism in the future.

In conclusion, we have analysed the role of non‐canonical NF‐κB signalling transcriptional regulator RelB for hepatocyte injury and liver fibrosis. And found that RelB is essential for the release of inflammatory factors and fibrogenic factors in damaged hepatocyte, and the progression of hepatic fibrosis.

## CONFLICT OF INTEREST

The authors declare that there is no conflict of interest.

## AUTHOR CONTRIBUTIONS

D. Zhou, W. Huang and J. Wei performed research and wrote the paper; J. Zhang, Z. Liu and M. Xiao analysed data; R. Ji, S. Ge and J. Wei raised animals and cultured cells; and Y. Fan and C. Lu designed research and wrote the manuscript.

## Data Availability

Data could be obtained upon request to the corresponding author.
